# Psychometric Properties of the Primary Care PTSD Screen for DSM-5: Findings From Family Members of Chinese Healthcare Workers During the Outbreak of COVID-19

**DOI:** 10.3389/fpsyt.2021.695678

**Published:** 2021-09-14

**Authors:** Peng Cheng, Nicholas Jasinski, Wanhong Zheng, Aradhita Yadava, Lirong Wang, Lingjiang Li, Lizhi Xu, Ying Zhou, Li Zhang, WeiHui Li

**Affiliations:** ^1^National Clinical Research Center for Mental Disorders, Department of Psychaitry, The Second Xiangya Hospital of Central South University, Changsha, China; ^2^Department of Behavioral Medicine and Psychiatry, School of Medicine, West Virginia University, Morgantown, WV, United States; ^3^Department of Biology and Psychology, Eberly College of Arts and Sciences, West Virginia University, Morgantown, WV, United States; ^4^XiangYa School of Medicine, Xiangya Hospital, Central South University, Changsha, China

**Keywords:** PTSD, psychometrics, assessment, PC-PTSD, cross-sectional study

## Abstract

**Background:** Minimal research has examined utility of PC-PTSD-5 in family members of frontline medical workers. The aims of our study were to develop and elucidate the psychometric properties of the Chinese version of the PC-PTSD-5 and to determine its usefulness in screening for possible PTSD in relatives of Chinese healthcare workers during the COVID-19.

**Methods:** We conducted a cross-sectional research in the relatives of medical staffs working in a general hospital during the COVID-19. Descriptive analysis was used to characterize demographic information of family members to find factors associated with PTSD symptoms. For reliability test, the internal consistency of PC-PTSD-5 was accessed using Cronbach's alpha coefficient. A validity test was assessed by Pearson's correlation between scales. A receiver operating characteristic (ROC) curve was used to evaluate the optimal cutoff score with the maximum Youden Index in this study.

**Results:** The result of demographic information indicated that gender and the type of work undertaken by medical staff in the family have a potential impact on the PTSD symptoms of medical staff's family members. Cronbach's alpha coefficient of PC-PTSD-5 was 0.83, indicating the high reliability. Good validity was also demonstrated by Pearson coefficient. By calculating the Youden index, a cutoff score of 2 was found to be optimal in our study, with sensitivity of 80.74% and specificity of 88.43%.

**Conclusions:** Our study has demonstrated the robust psychometric strengths of the PC-PTSD-5, introducing a reliable tool for screening PTSD among vulnerable and neglected families of these medical workers.

## Introduction

Post-traumatic stress disorder (PTSD) is a psychiatric disorder which is common in various public health emergencies. The key diagnostic criterion for PTSD is the onset of various behavioral and psychological symptoms, including nightmares, intrusive memories and re-experiencing of past traumatic events (i.e., flashbacks), hypervigilance, physiological hyperarousal, and anxiety after a traumatic event (1). PTSD is associated with various comorbidities including substance abuse, suicide, and depression and, as such, can have long-term adverse effects (2).

In reference to the ongoing COVID-19 outbreak, particularly during the initial phases in late-2019 and early-2020, the quick spread, high lethality rate, and lack of knowledge on effective treatment, the pandemic represented an unprecedented traumatic event for medical providers and their family members. Healthcare staff work against the epidemic in environments which are fast-paced and high-pressure, with high risk of infection of COVID-19, placing them in direct danger. This can disrupt family schedules, upset children, and cause worry and distress in spouses and relatives. Additionally, the current data was collected early in the pandemic, a time when details regarding route of transmission, transmission rate, and virus lethality were unknown, potentially increasing the distress for the healthcare staff and their families. When psychological difficulties present due to occupational traumas, the post-trauma reactions will certainly affect the wellbeing of their families. Additionally, due to the high risk of cluster transmission, lack of professional medical knowledge, and inadequate mental health care services it is speculated that the COVID-19 pandemic posed a particular threat to the family members of frontline medical staff which may have raised their risk for developing trauma-related symptoms.

Little has been documented academically about the possible immense psychological pressure that may have been experienced by their family members. Epidemiology studies about prevalence of PTSD in families were rarely seen. A systematic review analyzing the prevalence of PTSD symptoms during the COVID-19 pandemic, 7–53.8% of symptoms of PTSD were reported in the general population among various Asian and European countries during the COVID-19 (3). Another survey including frontline medical staff working in Wuhan during epidemic showed that PTSD symptoms in medical workers was as high as 31.6% (4). Based on this data and previous analyses, we speculated that the incidence of PTSD symptoms in family members of healthcare workers during the COVID-19 will be at a similar level.

In line with this speculation, previous studies showed that immediate relatives of individuals who have experienced a traumatic event have demonstrated increased risk for PTSD and associated symptoms. Kristensen et al. found that the incidence of PTSD among family members increased significantly when another member of the family died due to unnatural causes (5). A follow-up study of parents who lost children due to violent death indicated that 21% of mothers and 14% of fathers met criteria for PTSD 2 years after the death (6). After 5 years, the rate of mothers suffering from PTSD increased to 28% (7).

During the pandemic, the mental health conditions of family members of healthcare workers may often go unnoticed due to the acute crisis facing their family members on the frontline. This population requires greater academic attention, not only because of their morbidities but also from the healthcare workers' perspective. A survey of enrolled medical staff in Wuhan showed that “worry about family” is statistically related to the medical workers' psychological stress (8). Furthermore, an anonymous survey involving 4,618 medical professionals showed that good family relationships acted as an independent protective factor against distress (9). These results implied that good family relationship is crucial to the mental health of medical staff, which is an indispensable factor for their work efficiency. Good family relationship and the healthy mental state of family members are moderators of medical workers well-being. Therefore, investigating family members' psychological state is crucial for protecting the healthcare workers' mental well-being which may have a secondary benefit of improving the quality of medical and health services they provide, especially during the COVID-19 pandemic.

For screening for possible PTSD, two self-report instruments widely used in screening for PTSD are the Post-traumatic Stress Disorder Checklist for DSM-5 (PCL-5) (10) and the Primary Care Post-traumatic Stress Disorder Screen (PC-PTSD) (11). Both instruments are limited research was conducted on the psychometric property of PC-PTSD-5. Examples include studies on American veterans (12), PTSD patients, non-PTSD patients and healthy controls in South Korea (13), and Chinese children with traumatic exposure (14). These studies demonstrated suitable psychometric properties of the PC-PTSD-5 across populations with recommended cutoff scores ranging from 2 to 5.

However, the aforementioned studies have several limitations. First, the type of traumatic events was not filtered in the two studies conducted in Asia, leading to heterogeneity of patient samples which may not be generalizable to the family members of medical staff on the frontline of the COVID-19 pandemic. Second, most prior research did not restrict the participants' social backgrounds which directly impacted and influenced trauma-related symptoms. And third, many of the studies have indicated that people with different occupational backgrounds like rescue workers (15) and police officers (16) had obvious differences in the prevalence of PTSD due to factors such as work environment, social relationships, and occupational skills. From our review of the literature, no studies of possible PTSD in healthcare workers' families have been conducted, which is concerning given the aforementioned evidence that family well-being is vital to medical staff's mental health.

To fill the knowledge gap, the current study aimed to develop and elucidate the psychometric properties of the Chinese version of the PC-PTSD-5 by assessing its reliability and validity, and to determine its usefulness in screening for possible PTSD in family members of Chinese healthcare workers during the COVID-19 epidemic. The results will determine if the PC-PTSD-5 can be used as an efficient screening measure for PTSD in this population. Being able to quickly screen for individuals at-risk for PTSD in this population would be beneficial not only to the person being screened, but also the medical staff member related to them.

## Materials and Methods

### Participants and Study Design

This study was conducted as a companion to our published study in August 2020 (17). A convenience sample of family members of frontline medical staff from the Second Xiangya Hospital, affiliated with the Central South University in Changsha City, Hunan Province, China was the population in which our sample was drawn from. Family members were defined as relatives living with the frontline medical staff with high risk of infection of COVID-19. The data was collected from February 27th, 2020 to March 1st, 2020. The study site is a general hospital with a capacity of 3,500 beds. It was one of the designated hospitals by the Chinese government to admit febrile patients to rule out 2019-nCoV infection. The initial previous study sample of frontline workers is also described in full in our previous work (17). All participants were family members of these frontline medical residents or clinical lab specialists at a high risk of infection and psychological stress. An online questionnaire was chosen due to the impracticality of face-to-face interaction during the pandemic and the speed of data collection allowed by online administration. The speed of data collection was prioritized given the rapidly developing nature of the pandemic. We directly distributed a web-based, anonymous survey to all family members living with medical staff through social media applications (WeChat, QQ etc.). Details of the survey and its uses were provided to the participants *via* the survey software to ensure informed consent. Surveys had to be completed in their entirety for inclusion in the current analyses.

A total of 671 survey responses were obtained in our study. Demographic variables are provided in Table 1. This study was approved by the Ethics Committee of the Second Xiangya Hospital, Central South University.

**Table 1:** Demographic information and details about family members.

**Characteristic**	**Variable**	***N***	**Percent %**
Age (years)	<18	9	1.3
	18–25	69	10.3
	26–30	94	14.0
	31–40	182	27.1
	41–50	202	30.1
	51–60	115	17.1
Gender	Male	309	46.1
	Female	362	53.9
Educational level	Primary school	24	3.6
	Elementary school	97	14.5
	High school	109	16.2
	Junior college	96	14.3
	Undergraduate	267	39.8
	Master	54	8.0
	Doctor	24	3.6
Jobs of family members	Doctor	182	27.1
	Nurse	298	44.4
	Medical technician	191	28.5
Duration of family members worked on the first-line against COVID-19	<1 week	91	13.6
	1–2 weeks	97	14.5
	2–3 weeks	119	17.7
	3–4 weeks	119	17.7
	>4 weeks	245	36.5

### Measurements

#### PC-PTSD-5 Chinese Version

The PC-PTSD-5 is a five-item self-report screening measure as described in the introduction section. Items on this measure are scored dichotomously as either zero or one (0 = No; 1 = Yes). Users can estimate the risk of PTSD by calculating the total scores of five items in the scale. A cutoff score of three has been recommended as a reference point for effective screening for PTSD (12). PC-PTSD-5 Chinese version used in this study was translated from the original version of PC-PTSD-5.

We translated PC-PTSD-5 Chinese version by using translating-callback method (18). After the original version was translated into Chinese by two Chinese native speakers on the research team, the translation was then back-translated into English by two medical English specialists. The back-translation was compared with the original English version. Then a psychiatrist and two clinical psychologists reviewed and verified the accuracy of the translation. The Preliminary translation was modified until the back-translated version was comparable to the English version.

#### PCL-5

PCL-5 is a self-report measure based on the DSM-5 criteria for PTSD. It consists of 20 items divided into 4 subscales, corresponding to different symptom clusters in the DSM-5. Participants rate how much a problem described in the item statement bothered them over the past month on a five-point Likert scale from zero (not at all) to four (extremely). Items scores are summed to yield a total score ranging from 0 to 80. The psychometric properties of the Chinese version of this measure for use in frontline workers was documented in our previous publication (17). Given its strong validity as a screening measure of PTSD symptoms, it was used as the “gold standard” to determine the classification accuracy of the Chinese version of the PC-PTSD-5 used in the current study. Based on the recommended guidelines when using the PCL-5 (10), a score of 31 or above was used to determine the presence of possible PTSD.

#### General Anxiety Disorder-7

The GAD-7 is a seven-item screening measure used in assessing symptoms of generalized anxiety over the past 2 weeks (19). Individuals are asked to rate how frequently they experience the symptoms described in the item statement, rating it using a four-point Likert scale ranging from zero (not at all) to three (nearly every day). The GAD-7 has been widely used in China and the reliability and validity of the Chinese version of GAD-7 has been confirmed (20). We used this brief scale to assess global anxiety and to explore the discriminant validity of Chinese version of the PC-PTSD-5.

#### Patient Health Questionnaire-2

The PHQ-2 is a widely used, efficient, and simple two-item assessment for depression experienced over the past 2 weeks (21). Responses are rated on a four-point Likert scale (0 = Not at all, 3 = Nearly every day). PHQ-2 has been demonstrated as a reliable and valid screening tool for depressive symptoms in China (22). Results from the PHQ-2 were also used to examine the discriminant validity of the Chinese version of the PC-PTSD-5.

#### Perceived Stress Scale-10

The PSS-10 is a widely-used 10-item self-report measure with established reliability and validity in measuring levels of current stress (23). A review of the psychometric evidence of the PSS-10 showed that the PSS-10 is an easy-to-use questionnaire with established acceptable psychometric properties. The psychometric properties of the PSS-10 have also been examined among various populations including college students and police officers in China (24, 25). Results from the PSS-10 were used to explore the relationship between current stress level and scores on the Chinese version of the PC-PTSD-5 in the sample population.

#### 10-Item Connor–Davidson Resilience Scale

The CD-RISC-10 is a 10-item self-report measure used to assess resilience defined as the ability to cope with adversity (26). The reliability and validity of the scale has been tested for earthquake victims (27), depression patients and college students in China (28). Results from the PSS-10 were also used to explore the relationship of resilience and scores on the Chinese version of the PC-PTSD-5 in the sample population.

### Data Analysis

Statistical analyses were performed with SPSS version 25.0 (IBM Corp. New York, USA.) and MedCalc version 19.6 (MedCalc Software Ltd. Ostend, Belgium.). Descriptive analysis was used to characterize the study sample in terms of demographic information and including the duration their family member was working on the frontline treating COVID-19. Reliability was evaluated by determining the internal consistency of the Chinese version of the PC-PTSD-5 using Cronbach's alpha coefficient, with a minimum result of 0.70 considered satisfactory (29). Convergent and divergent validity were evaluated using the Pearson correlation coefficient (PCC; Pearson's *r*). To evaluated classification accuracy further, receiver operating characteristic (ROC) analyses were conducted for the PC-PTSD-5 and the PCL-5. Sensitivity, specificity, positive predictive value, negative predictive value, and likelihood ratios for the Chinese version of the PC-PTSD-5 were calculated using the PCL-5 as the “gold standard” for classification accuracy. Recommended cut-offs of ~90% specificity were calculated for the PC-PTSD-5 to minimize false positive misclassifications. Summarizing classification accuracy through a single numeric value was examined by calculating the Youden Index value (*J* = sensitivity + specificity – 1) (30). Perfect accuracy is defined as *J* = 1 whereas *J* = 0 suggests agreement purely due to chance.

## Results

### Demographic and Descriptive Statistics

We summarized the participants' characteristics in Table 1. A total of 671 family members of medical staff were included in this study. The sample was majority female (53.9%). The largest age group in the sample was aged 41 to 50 (30.1%) with nearly 60% of the sample ranging in age from 31 to 50. Approximately 61% of the sample had some college experience or higher. A majority of the sample had family members classified as nurses or medical technicians (72.9%) with the rest having family members classified as medical doctors. A majority of the sample had family members that had worked <4 weeks on the frontlines of the COVID-19 pandemic (63.5%). It should be noted that major media outlets and governmental organizations document the start of the pandemic in Wuhan, Hubei province, China as approximately December 2019 to January 2020. The current data was collected in late-February to early-March 2020. As such, the lack of longer-term exposure (i.e., >4 weeks) to frontline work at the time of data collection was to be expected.

Means, standard deviations, minimum, and maximum values for the PC-PTSD-5, PCL-5, GAD-7, PHQ-2, PSS-10 and CD-RISC-10 are shown in Table 2. The mean scores on the PCL-5 and the PC-PTSD-5 were, respectively, 19.54 (SD = 21.45) and 1.37 (SD = 1.64). The typical cutoff for possible diagnosis of PTSD using the PCL-5 is 31 (17) and 20.1% of the current sample scored above this threshold. Analysis of variance indicated that PC-PTSD-5 score differed significantly by gender (men>women), and PCL-5 score differed significantly by gender (men > women) and the occupation of frontline medical workers (Doctor>Nurse>Medical technician). Detailed data has been shown in Table 3.

**Table 2:** Normative data for the psychological assessments.

**Scale**	**M**	**SD**	**Possible range**	**Observed range**	**Cronbach's alpha**
PC-PTSD-5	1.37	1.64	0–5	0–5	0.83
PCL-5	19.54	21.45	0–80	0–79	0.91
GAD-7	4.88	4.57	0–21	0–21	0.92
PHQ-2	1.14	1.35	0–6	0–6	0.81
PSS-10	15.92	5.99	0–40	0–40	0.81
CD-RISC-10	26.22	9.05	0–40	0–40	0.96

**Table 3:** Analysis of variance for score of two measurements of PTSD.

**Demographic variable**	**PC-PTSD**		***F***	***P***	**PCL-5**		***F***	***P***
	***M***	**SD**			***M***	**SD**		
Age (years)			1.18	0.32			1.139	0.35
<18	2.56	1.88			22.56	29.93		
18–25	1.49	1.8			21.96	26.2		
26–30	1.46	1.65			22.69	22.15		
31–40	1.31	1.63			17.05	18.33		
41–50	1.32	1.62			19.77	21.87		
51–60	1.32	1.57			18.79	20.75		
Gender			5.22	**0.02**			8.076	**<0.01**
Male	1.53	1.72			22.07	23.09		
Female	1.24	1.56			17.38	19.71		
Educational level			0.25	0.96			0.76	0.60
Primary school	1.21	1.74			20.08	24.896		
Elementary school	1.46	1.71			22.04	23.372		
High school	1.28	1.6			16.2	19.415		
Junior college	1.28	1.59			19.75	20.797		
Undergraduate	1.39	1.61			20.19	21.377		
Master	1.44	1.8			17.83	21.582		
Doctor	1.54	1.82			19.79	22.177		
Jobs of family members			1.48	0.24			5.58	**<0.01**
Doctor	1.46	1.81			21.8	25.34		
Nurse	1.43	1.51			20.92	20.09		
Medical technician	1.2	1.67			15.22	18.79		
Duration of family members worked on the first-line against COVID-19			0.57	0.68			1.648	0.16
<1 week	1.21	1.68			18.37	21.96		
1–2 weeks	1.57	1.81			15.31	18.24		
2–3 weeks	1.38	1.62			20.41	22.56		
3–4 weeks	1.36	1.63			22.52	21.62		
>4 weeks	1.36	1.59			19.77	21.67		

*Categories with statistically significance were marked in bold*.

### Internal Consistency Reliability

Cronbach's alphas were calculated for the internal consistency of PC-PTSD-5. The Cronbach's alpha coefficient of the total scale was 0.83, exceeding the 0.70 level and demonstrating the high reliability of the PC-PTSD-5 Chinese version in our sample. In comparison, a previous PC-PTSD-5 study conducted in a sample of Chinese children obtained a Cronbach's alpha of 0.47 (14), indicating greater consistency in our sample.

### Convergent Validity and Discriminant Validity

PCCs (Pearson *r*) between PCL-5, GAD-7, PHQ-2, PSS-10, CD-RISC-10 and PC-PTSD-5 are shown in Table 4. There was a significant and strong correlation (31) between the Chinese version of the PC-PTSD-5 and PCL-5 (*r* = 0.754, *p* < 0.01) in our sample suggesting adequate convergent validity. Meanwhile, measures used to asses syndromes and psychological variables other than PTSD, including GAD-7, PHQ-2 and CD-RISC-10, were weakly correlated (31) with the PC-PTSD-5 (*r* = −0.196, 0.189, and 0.209, respectively, all *p* < 0.01) providing strong evidence for divergent validity. The PSS-10 and PC-PTSD-5 not were significantly correlated as well.

**Table 4:** Correlations between PCL-5, GAD-7, PHQ-2, PSS-10, CD-RISC-10 and PC-PTSD-5.

**Assessment category**	**PC-PTSD-10**	**PCL-5**	**GAD-7**	**PHQ-2**	**PSS-10**	**CD-RISC-10**
PC-PTSD-5	1	0.754^**^	0.189^**^	0.209^**^	−0.021	−0.196^**^
PCL-5	0.754^**^	1	0.201^**^	0.205^**^	−0.009	−0.174^**^
GAD-7	0.189^**^	0.201^**^	1	0.692^**^	0.368^**^	−0.182^**^
PHQ-2	0.209^**^	0.205^**^	0.692^**^	1	0.319^**^	−0.265^**^
PSS-10	−0.021	−0.009	0.368^**^	0.319^**^	1	0.248^**^
CD-RISC-10	−0.196^**^	−0.174^**^	−0.182^**^	−0.265^**^	0.248^**^	1

** *P < 0.01*.

### ROC Analysis

**Figure 1:**
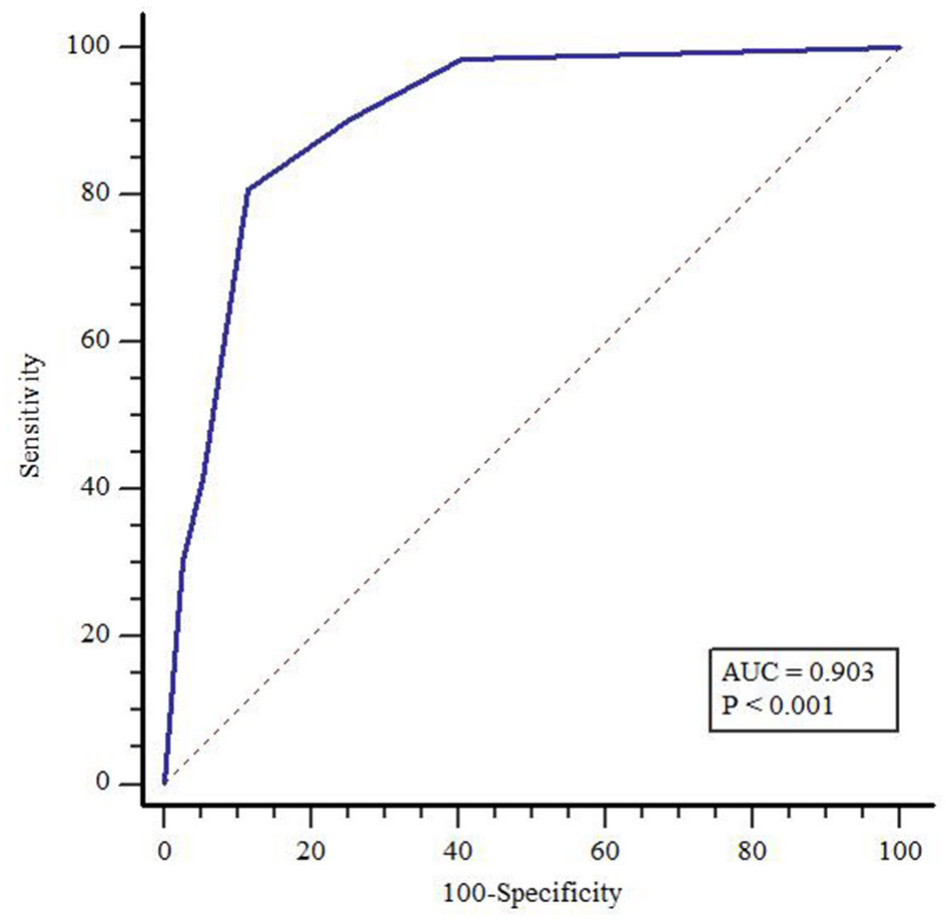
Receiver operating characteristic (ROC) curves for the PC-PTSD-5.

Using the cutoff of 31 or greater on the PCL-5, a total of 135 people (20.1%) were classified as possible PTSD with 536 people (79.9%) falling in the non-clinical range. Table 5 presents the diagnostic efficiency statistics for the PC-PTSD-5 in our sample. Maintaining a specificity of 0.90 is commonly accepted as the minimum required for use in psychological assessment. At this threshold, a cutoff score of 3 on the PC-PTSD-5 for family members would be required, though sensitivity at this score is low (41.48%). A cutoff score of 2 reduced specificity to 88.43% but improved sensitivity to 80.74% and was the optimal score based on the Youden index calculation. According to the result of ROC analysis shown in Figure 1, the area under the curve (AUC) obtained for the PC-PTSD-5 was 0.903 (95% CI: 0.878–0.924) suggestive of excellent accuracy in identifying possible PTSD in our sample.

**Table 5:** Operating characteristics of the PC-PTSD-5.

**Cutoff score**	**Sensitivity %**	**Specificity %**	**PPV %**	**NPV %**	**+LR**	**-LR**	**Kappa**	**AUC**
>0	98.52	59.33	37.9	99.4	2.42	0.025	0.362	0.903
>1	90.37	74.63	47.3	96.9	3.56	0.13	0.485	
>2	80.74	88.43	63.7	94.8	6.98	0.22	0.629	
>3	41.48	94.59	65.9	86.5	7.67	0.62	0.419	
>4	30.37	97.39	74.5	84.7	11.63	0.71	0.357	

## Discussion

Our study aimed to elucidate the psychometric properties of Chinese version of PC-PTSD-5 in the sample of family members of medical staff working on the frontline during a short timeframe in the early months of the COVID-19 pandemic-19. To our understanding, this is the first study related to the PC-PTSD-5 reliability, validity, and diagnostic accuracy in the context of a large-scale public emergency enrolling the family members of medical staff. Our study demonstrated that PC-PTSD-5 can be applied as a reliable screening measure of PTSD symptoms in families of medical staff during COVID-19.

Based on our previous study regarding the reliability and validity of PCL-5 during COVID-19, we used it as the “gold standard” measure of PTSD to evaluate the application of PC-PTSD-5 in this sample. We also analyzed the Cronbach's coefficient of PC-PTSD-5 and its Pearson correlation with other scales to test its reliability and validity. Finally, we used ROC analysis to find the best cutoff providing the highest degree of sensitivity and specificity for the diagnosis of PTSD. Overall, the Cronbach's α of the PC-PTSD-5 was 0.83, which is considered to be evidence of strong reliability (Cronbach's α > 0.7) (32). The PC-PTSD-5 had strong correlations with the PCL-5 and significant, but weak correlations with measures of generalized anxiety, depression, and perceived stress suggesting strong evidence of convergent and divergent validity.

For ROC analysis, AUCs > 0.71 is considered to have large effect sizes (33). Our ROC analysis found that total scores on the PC-PTSD-5 had a large effect, with an AUC of 0.903 (95% CI: 0.878–0.924). A previous study conducted in Korea regarding the diagnostic characteristics of PC-PTSD-5 obtained an AUC of 0.898 (13), which lends further support for the diagnostic accuracy of the PC-PTSD-5.

A range of the cutoff values was tested for effective discrimination of possible PTSD among our sample. The calculated Youden index suggested that 2 points was the optimal cutoff value. Previous studies in other samples (12, 34) indicated that 3 was the suggested cut-off value for PC-PTSD-5. In our sample, when a cutoff score of 3 was applied specificity was above 0.90 (0.945), the level typically suggested for use in psychological assessment. However, at this cutoff, sensitivity was low (41.48%). When a cutoff score of 1 was used in this study, sensitivity was high (0.904) and specificity was low (0.746).

The lower optimal cutoff score in our sample when compared to previous research with clinical patient and veteran populations is likely due to sample characteristics. Previous studies in these populations had higher levels of reported symptoms and a larger number of individuals with PTSD. In our sample 20% met criteria for possible PTSD as defined by positive findings on the PCL-5. Given the lower base rate of possible PTSD in our sample, a lower cutoff score would be expected to ensure an adequate sensitivity-specificity ratio.

Ultimately the choice of appropriate cutoff value should be determined based on the goal of assessment. If using the PC-PTSD-5 as a screening measure to determine the necessity for further follow-up and treatment, sensitivity would be of greater importance to ensure the highest percentage of at-risk individuals was captured. Based on our data, a score of 1 or 2 would suggest the need for further assessment. However, if the PC-PTSD-5 were to be used in helping to determine diagnosis for PTSD, higher specificity would be valued to avoid false-positives. In this case, the minimum cutoff to be used would be 3 to maintain adequate specificity. However, caution is recommended in using the PC-PTSD-5 for solely diagnostic purposes as sensitivity is low at the cutoff of 3 indicating that many individuals with possible PTSD would be missed.

The result of this study demonstrated the psychometric robustness of PC-PTSD-5 in this sample of family members of medical staff on the frontline of the COVID-19 pandemic. Nevertheless, possible PTSD-related symptoms in family members of other occupations with high pressure and an increased risk for exposure to adverse or traumatic incidents, such as police and firefighters, are worth further study. A study involving 500 Indian police officers found that the high-stress nature of police work is an important cause of family conflict (35). A study of American firefighters also found that work-family conflict and stress were moderately correlated (36) suggesting that the stressful nature of one's work might transfer distress onto family members. Due to the possible similarity of work-stress issues faced by families of these populations and families of healthcare workers, this study provides preliminary evidence of the usefulness of the PC-PTSD-5 in screening family member for possible PTSD. Our study tried to provide a model for future research to evaluate the performance and usefulness of the PC-PTSD-5 in these other, potentially similar populations.

## Limitations

Despite the important findings of this study, there are limitations which warrant discussion. Firstly, our sample lacked the direct exposure of traumatic events like experiences confronted by their family relative who was a healthcare worker. It means that not all participants in our study might fully meet all criteria concerning criterion A of PTSD diagnosis based on DSM-5. The lack of direct experiences of traumatic events in our sample restrain the application of our results to some degree. Future psychometric study of PC-PTSD-5 in the sample with direct traumatic experiences met the criterion A is necessary. Secondly, constrained by the highly contagious nature of the COVID-19, we adopted a web-based questionnaire to collect data, so we were unable to use the Clinician administered PTSD scale for DSM-5 (CAPS-5) as the diagnostic standard. Although our previous research has verified that PCL-5 has good reliability and validity in this pandemic, due to the importance of CAPS-5 in the diagnosis of PTSD (10), replication with a more comprehensive assessment of PTSD would be beneficial. Thirdly, given the stigma of mental health issues in China (37), it is possible that respondents may have been hesitant to acknowledge trauma-related symptoms during the survey. However, it is also possible that the anonymous nature of the survey may have ameliorated this stigma-bias, leading to a more accurate capturing of these stigmatized symptoms. Fourthly, this study collected survey data from the families of all front-line medical staff, but without classifying according to specific departments or level of risk exposure, making more fine-grained analysis difficult. Finally, further validation of the PC-PTSD-5 for use in family members of high-stress job holders in other countries will also be necessary to recommend its use with other cultures and ethnicities.

## Conclusions

Our study has demonstrated the robust psychometric strengths of the PC-PTSD-5 when used with family members of medical staff during the COVID-19 pandemic. It introduces a reliable tool for screening PTSD among vulnerable families of these medical workers.

## Data Availability Statement

The datasets presented in this article are not readily available due to privacy reasons. Requests to access the datasets should be directed to WeiHui Li, weihui_li@csu.edu.cn.

## Ethics Statement

The studies involving human participants were reviewed and approved by the Ethics Committee of the Second Xiangya Hospital, Central South University. Written informed consent to participate in this study was provided by the participants' legal guardian/next of kin.

## Author Contributions

PC: data collection, literature review, and manuscript drafting. LW, LX, and YZ: managed the ethical review process. NJ, WZ, AY, LL, LZ, and WL: manuscript drafting and revision. All authors read and approved the final manuscript.

## Funding

This study was supported by Hunan Provincial Natural Science Foundation of China (No. 2018JJ2592) and Hunan Key Research and Development Program (No. 2018SK2136).

## Conflict of Interest

The authors declare that the research was conducted in the absence of any commercial or financial relationships that could be construed as a potential conflict of interest.

## Publisher's Note

All claims expressed in this article are solely those of the authors and do not necessarily represent those of their affiliated organizations, or those of the publisher, the editors and the reviewers. Any product that may be evaluated in this article, or claim that may be made by its manufacturer, is not guaranteed or endorsed by the publisher.
