# Supplementary material for: Psychometric Properties of the Primary Care PTSD Screen for DSM-5: Findings From Family Members of Chinese Healthcare Workers During the Outbreak of COVID-19
Source: Front Psychiatry. 2021 Sep 14;12:695678. doi: 10.3389/fpsyt.2021.695678 (PMC8477025; doi:10.3389/fpsyt.2021.695678)
Supplement: Supplementary file 1 [file Data_Sheet_1.PDF]

Primary Care PTSD Screen for DSM-5 (PC-PTSD-5)

Since the beginning of COVID-19, have you ever felt frightened, horrible or traumatic caused by the COVID-19 or related factors?	
If your answer is 'yes', please continue with this screening.	
In the past month, have you…	YES / NO
1. Had nightmares about the event(s) or thought about the event(s) when you did not want to?	
2. Tried hard not to think about the event(s) or went out of your way to avoid situations that reminded you of the event(s)?	
3. Been constantly on guard, watchful, or easily startled?	
4. Felt numb or detached from people, activities, or your surroundings?	
5. Felt guilty or unable to stop blaming yourself or others for the event(s) or any problems the event(s) may have caused?	
